# Age-Related Decline of Gastric Secretion: Facts and Controversies

**DOI:** 10.3390/biomedicines13071546

**Published:** 2025-06-25

**Authors:** Francisco Vara-Luiz, Ivo Mendes, Carolina Palma, Paulo Mascarenhas, Gonçalo Nunes, Marta Patita, Jorge Fonseca

**Affiliations:** 1 Gastroenterology Department, Hospital Garcia de Orta, 2805-267 Almada, Portugal; 2 Aging Lab, Egas Moniz Center for Interdisciplinary Research (CiiEM), Egas Moniz School of Health and Science, 2829-511 Almada, Portugal

**Keywords:** aging, gastric secretion, chronic atrophic gastritis, *Helicobacter pylori*, precision medicine

## Abstract

Aging is associated with structural and functional changes in the gastrointestinal tract; however, its impact on gastric secretion remains unclear. This scoping review examines whether gastric secretion declines with age and explores its clinical implications. Following the PRISMA guidelines, PubMed, Web of Science, Embase, and Google Scholar were systematically searched from inception to December 2024. Fifteen studies (both animal and human) met the inclusion criteria: they were written in English, directly relevant to aging and gastric secretion, and had a clearly stated methodology. Evidence strength was assessed using the GRADE framework, revealing predominantly low to moderate certainty due to small sample sizes and observational study designs. Animal studies have demonstrated reduced acid secretion in older rats, which is attributed to mucosal atrophy and diminished responsiveness to gastrin. Recent human studies suggest that aging does not directly reduce acid output, as reduced acid secretion may result from a higher prevalence of atrophic gastritis, *Helicobacter pylori* infection, and the widespread use of proton pump inhibitors. Antisecretory therapy may lack benefits in older adult patients with hypochlorhydria/achlorhydria and increase the risk of adverse effects. Pepsin output declines with aging due to reduced chief cell function, although its clinical impact on digestion is unclear. Since intrinsic factor secretion far exceeds the amount necessary for its physiological function, even low amounts seem to be sufficient to prevent cobalamin deficiency. Age-related decline in gastric secretion is mostly attributed to age-associated disorders; however, impairment of secretory function in older people is frequent. Future research should prioritise longitudinal studies, larger cohorts, and histology-stratified analysis.

## 1. Introduction

Society is witnessing a remarkable increase in life expectancy, resulting in an aging population worldwide [1]. Healthcare providers require a basic understanding of essential aging medicine to provide adequate healthcare to older patients [2]. However, current knowledge about digestive changes in the older population is scarce, as most clinical trials exclude older patients [3].

Normal physiology of the stomach is required for digestion, absorption of nutrients and drugs, and protection against ingested pathogens, as the gut is the largest human surface in contact with environmental factors. Aging is associated with structural and functional decline in all organs and systems, including the digestive system. Indeed, previous studies have documented several structural and functional changes in the gastrointestinal tract [4,5], as up to 80% of older individuals have morphological changes in their stomachs [6]. The stomach has an important secretory function [7], producing pepsinogen for primary protein digestion, hydrochloric acid for protection, and intrinsic factor (IF) for the uptake of cobalamin.

Despite a progressively aging population, the influence of age on gastric secretion remains a matter of debate, with several studies from different research teams reporting conflicting results [8,9,10]. Moreover, the impact of sex, smoking status, geography, and metabolic and clinical factors has not been clearly determined.

Due to multimorbidity and the lack of deprescribing procedures, older adult patients are particularly prone to polypharmacy [11]. Proton pump inhibitors (PPIs) are among the most prescribed drugs for the treatment of heartburn, gastroesophageal reflux disease, and peptic ulcer disease, as well as for the concomitant treatment of *Helicobacter pylori* (*H. pylori*) infection [12]. Furthermore, PPIs are frequently prescribed for “gastric protection” in older adults undergoing polypharmacy. However, data on a possible age-related decline in gastric secretion may show that PPIs and other antisecretory therapies are ineffective and inappropriate in these patients.

By addressing gaps in knowledge and suggesting future exploration, this review aims to contribute to (1) assessing the influence of aging on gastric secretion, (2) evaluating the importance of gastric secretion of factors frequently associated with aging, and (3) discussing the potential clinical impact of these findings.

## 2. Materials and Methods

The protocol for this scoping review was defined a priori by all authors and registered at the International Platform of Registered Systematic Reviews and Meta-Analysis (INPLASY—ID Number INPLASY202560078). We prepared our review design following the Preferred Reporting Items for Systematic Reviews and Meta-Analyses (PRISMA) extension for scoping reviews (Table S1) [13] and assessed the evidence quality using the GRADE framework. The primary research question guiding this study was as follows: Does gastric secretion decrease with age? A comprehensive search of four electronic databases (PubMed, Web of Science, Embase, and Google Scholar) was conducted until December 2024. Given the paucity of recent studies, no restrictions were applied to the publication dates of the sources. The integration of keywords and subject headings was conducted following the thesaurus of each database, employing the following search strings: (Aging OR elder OR older OR geriatric) AND (1) (gastric secretion) OR (2) (hydrochloric acid OR gastric acid) OR (3) (intrinsic factor) OR (4) pepsin OR pepsinogen). A manual search of the reference lists of the studies included in the final analysis was conducted to identify any additional relevant literature. A tripartite group of authors (FVL, IM, and CP) conducted an electronic search of the database. The final decision for inclusion was made according to the following criteria: studies must be published in English or another language with an accompanying English abstract. The sample must be composed of either animal models or humans. The articles must address the possible impact of aging on gastric secretion. Articles lacking English abstracts or failing to meet the inclusion criteria were excluded. In order to fulfil the requirements of the article selection task, a preliminary evaluation of the titles and abstracts was conducted to ascertain the extent to which there was consensus regarding their inclusion and/or exclusion under the predefined criteria. In instances where the title and abstract were either uninteresting or inconclusive, the entire document was read to mitigate any potential loss of valuable information that might have occurred during the study. The articles selected for inclusion in this study were based on their relevance to the topic. A predefined table extracted the necessary data from each eligible study, listing the author’s last name, publication year, study characterisation (animal or human studies), number of patients (younger and older groups, if applicable), outcomes in gastric secretion, and general comments that could influence the generalisation.

To account for the correlation of multiple arms within the same study, a multilevel meta-analysis was conducted using a random-effects model with a nested structure (‘~1|Study/Author’) implemented via the ‘rma.mv()’ function in the metafor R package (version 4.8-0). The outcome variable was the mean basal acid output (mEq/h), and the variance was based on the reported standard deviation and sample size. A meta-regression was also performed using age (mean per study group) as a continuous moderator in the same multilevel framework. Between-study heterogeneity was quantified using the restricted maximum likelihood (REML) estimator. A forest plot was generated from the multilevel model to display the unadjusted means and confidence intervals. Predicted (age-adjusted) values were derived from the regression model and visualised in a separate forest plot that included a pooled estimate based on a secondary meta-analysis of the predicted means. This adjusted forest plot corresponds approximately to the median age of the included population, which was 50 years. A bubble plot of the meta-regression was constructed, scaling points by study weight and including both a fitted regression line and the 95% prediction interval.

## 3. Results

**Figure 1:**
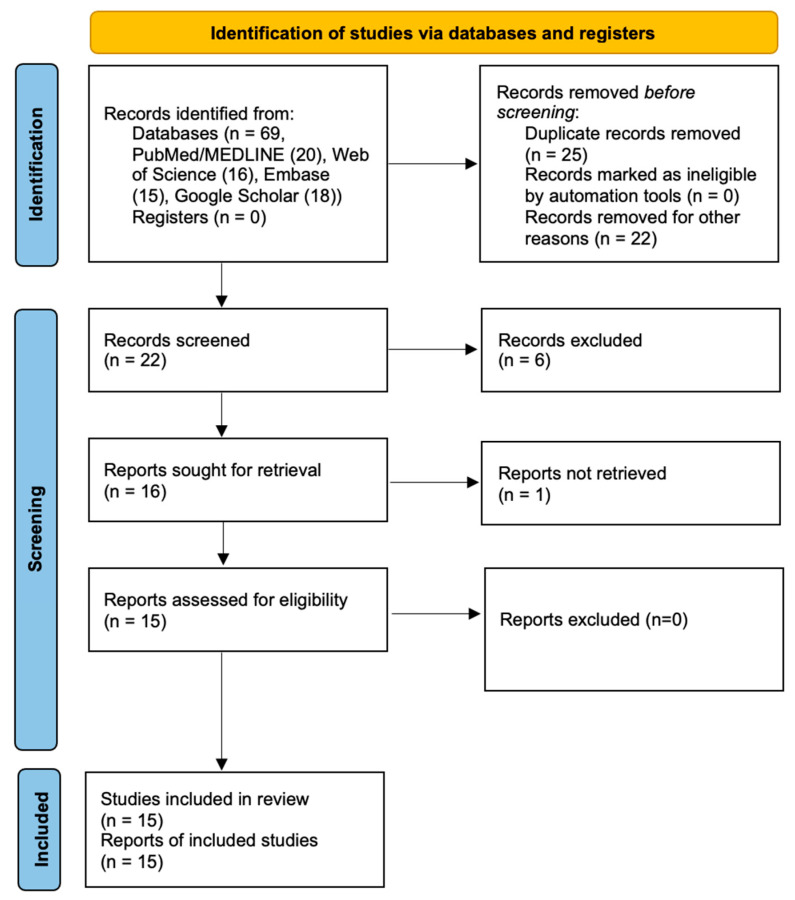
PRISMA flow diagram representing the research pathway.

The search conducted in the aforementioned databases yielded 69 articles. After the elimination of 47 articles, of which 25 were duplicates, and 22 did not meet the inclusion criteria, 22 articles were screened. The respective titles and abstracts of these articles were then extracted for analysis. After thoroughly examining the titles and abstracts of the remaining articles, six were disqualified because they did not align with the subject matter of the study. A total of 16 articles were retrieved and selected for full-text analysis. However, it was not possible to retrieve the 16th article, which consequently prevented the full reading of the text. Following a thorough and meticulous selection process, 15 articles were included in the present scoping review. A flow chart illustrating the research pathway is presented in Figure 1, employing the PRISMA flow diagram [13].

The 15 eligible articles were read in full, and their content was analysed to meet the goals proposed in this scoping review. The features and main findings of the available evidence regarding the influence of aging on gastric secretion are summarised in Table 1 in descending chronological order of publication.

**Figure 2:**
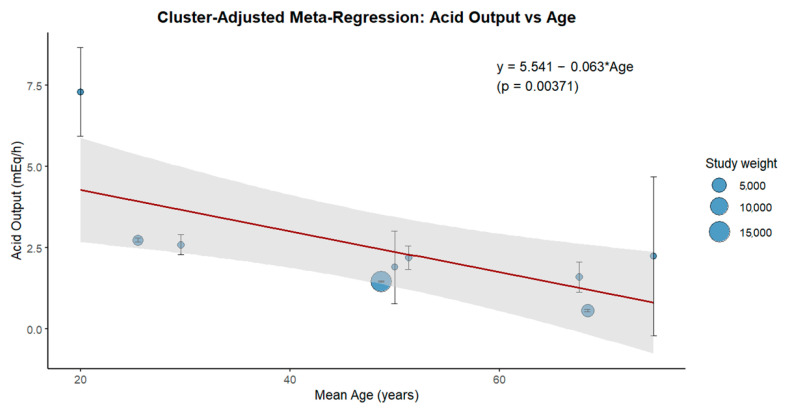
Bubble plot of the cluster-adjusted meta-regression model showing the association between age and basal acid output. Each point represents a study arm scaled by the inverse of its variance (study weight). The red line shows the fitted regression (slope = −0.063; *p* = 0.0037), and the gray shaded area represents the 95% prediction interval.

The clustered multilevel meta-analysis yielded a pooled estimate of the mean acid output of 2.46 mEq/h (95% CI: 1.26 to 3.65), with considerable heterogeneity observed among study arms due to age differences and variation within the studies. Meta-regression (Figure 2) revealed a statistically significant negative association between age and acid output (slope = −0.063; *p* = 0.0037), with the regression equation:

Acid Output = 5.541 − 0.063 × Age

This supports the hypothesis that gastric acid secretion declines with advancing age. The 95% prediction interval around the regression line also illustrates this decline across the age range.

**Figure 3:**
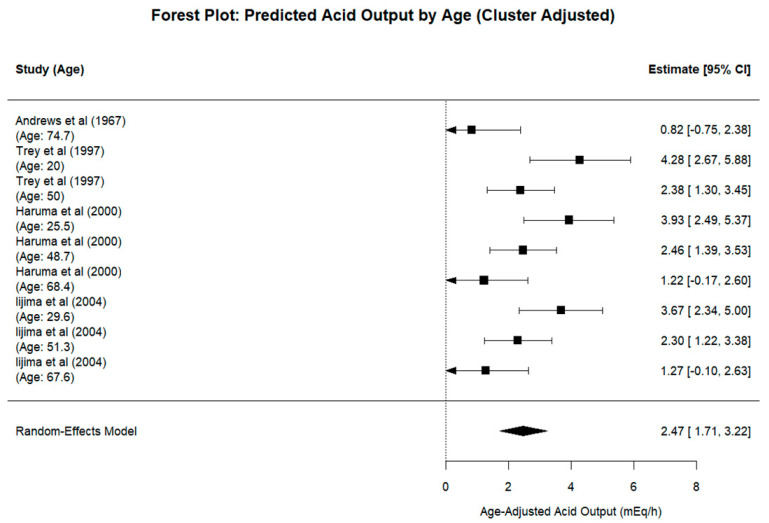
Cluster-adjusted forest plot of age-adjusted predicted acid output (mEq/h) [8,20,22,24]. Predicted values were obtained from a multilevel meta-regression model, including age as a continuous moderator. All estimates were standardised to the median age of the included groups (50 years). The pooled estimates and 95% confidence intervals are shown at the bottom.

The forest plot of age-adjusted predicted values (Figure 3) showed a pooled mean of 2.47 mEq/h (95% CI: 1.71 to 3.22). The use of a multilevel model appropriately accounts for the non-independence of the study arms and improves the precision in estimating both pooled means and moderator effects.

## 4. Discussion

### 4.1. Impact of Aging on Gastric Secretion

Several animal and human studies have investigated the influence of aging on gastric secretion. The former includes mainly Fischer 344 rats, as they have been previously studied for morphological changes in major organs with aging. Their gastrointestinal tract has been shown to be remarkably free of lesions, making them a reliable model of the aging gastrointestinal tract [25]. Evidence quality, assessed using the GRADE framework, revealed predominantly low to moderate certainty due to small sample sizes and observational designs.

#### 4.1.1. Animal Model Studies

Khalil et al. [6] demonstrated that basal- and gastrin-stimulated gastric acid secretion and serum and antral gastrin concentrations are significantly reduced in older rats. A similar observation was made by other researchers, suggesting that aging lowers the gastric mucosal capacity to secrete acid and pepsin. This phenomenon may be partly attributed to mucosal atrophy, as mucosal weight and DNA/RNA content were significantly lower in older rats than in their younger counterparts [14]. In addition to the decrease in serum gastrin levels, exogenous administration of pentagastrin does not stimulate gastric mucosal growth in older rats [26,27]. These animal studies led to the postulation that the lack of responsiveness of the parietal and chief cells to the secretory and growth-promoting actions of gastrin could be the result of age-associated loss of its receptor expression in the gastric mucosa [28] and a higher ratio of somatostatin to gastrin cells in the antral mucosa [29]. No animal studies have investigated the influence of aging on IF secretion.

#### 4.1.2. Human Studies

Earlier human studies have demonstrated a progressive decrease in acid secretion with age [8,15,16,30]. Goldschmiedt et al. [9] challenged this concept prospectively because they found a higher basal acid output, meal/gastrin-stimulated acid secretion, and peak acid output in older males and females. However, the differences between younger female subjects were not significant. The authors also postulated that increased acid secretion with aging may contribute to the age-related increase in ulcer incidence in Western countries. However, this study has two limitations. First, the sample size was small (less than 50 patients. Second, the ages of the patients ranged from 44 years to 65 years (mean age: 57 years). In developed societies, the chronological age at which a citizen is considered an older adult is usually 65 years. All these issues limited the conclusions, and it is possible that these findings would not persist in an older group of subjects (≥65 years old).

Feldman et al. [18], in line with a Finnish study [17], postulated that if patients with chronic atrophic gastritis (CAG) are excluded, gastric acid secretion would not decline with aging, as demonstrated by a regression model showing no independent effect of age. Instead, the high prevalence of gastritis may explain the reduced secretion in older adults. A different observation was noted concerning pepsin output, which was statistically reduced in the elderly group and seemed to have been caused, in large part, by aging. These findings suggest that chief cell mass/function declines with normal aging. Another study corroborated these observations [19] and showed that acid secretion did not differ between older and younger individuals, although pepsin secretion was not evaluated. Moreover, age-related achlorhydria has not been found in other studies of patients with gastrointestinal symptoms [31] or small cohorts of healthy subjects [32,33]. In this regard, the typical consequences of hypochlorhydria/achlorhydria (e.g. gut colonisation by enteric pathogens and malabsorption of drugs and nutrients) have not been reported with a higher prevalence in the older population. Shih et al. [23] retrospectively evaluated ambulatory pH in patients with gastrointestinal comorbidities that were not excluded from the final analysis. They found no change in gastric acid secretion with age, emphasising that elderly patients without atrophic gastritis retained their ability to produce gastric acid.

The Japanese population has a high incidence of gastric cancer [34], and acid secretion is believed to decline sharply with aging. Indeed, acid secretion has been shown to decline in H. pylori-infected individuals. However, the results in patients without *H. pylori* infections are inconsistent [21,22,24,35].

Although there is a generalised conviction that older adults are at risk of altered production of IF [36], only a few studies have evaluated the possible influence of aging on its secretion. Andrews et al. [8] showed a parallel decline in acid output and IF secretion with aging. However, despite the small sample size, all but one patient had some degree of CAG. This observation makes it difficult to assess whether the observed reduction in gastric secretion results from aging, chronic gastritis, or mucosal atrophy.

We must acknowledge some limitations of the studies on gastric secretion in older patients. Earlier studies had a variable definition of “elderly,” ranging from >40 years to >70 years of age [9,18]. In addition, these studies were limited by small sample sizes, ranging from 11 to 62 individuals [9,10,18,20]. This likely reflects the invasiveness of the procedures, such as the placement of a nasogastric tube. More recent studies, including those with ambulatory pH monitoring [23] and the use of a quinine-containing exchange resin that is ingested and then excreted in the urine [19], may help overcome these limitations. Moreover, none of the analyzed works considered the potential impact of dental conditions in the recruited patients, which are significant predictors of nutritional status and may also indirectly influence gastric secretion [37,38].

### 4.2. Impact of Other Factors on Gastric Secretion

#### 4.2.1. Sex, Smoking Factors and Oral Health

Most previous studies have reported higher gastric acid secretion in males than in females [9,17], which has been ascribed to variations in gastric surface area [17] or differences in parietal cell sensitivity to gastrin [39]. In contrast, the female sex hormone oestradiol inhibits acid secretion [40], which may be associated with increased acid secretion in post-menopausal females, as outlined in a Finnish study [17]. However, when total lean body weight and smoking habits are considered, these sex differences disappear [18]. Smoking is an independent risk factor for peptic ulcer disease [41], and a study with a small number of patients suggested higher gastric secretion in smokers [18]. Possible mechanisms include a nicotine-mediated increase in parietal and chief cell mass and a reduction in prostaglandin E2, an endogenous inhibitor of gastric secretion [42].

Food intake is a major physiological stimulus for gastric secretion. During meal ingestion, maximal acid secretion is achieved (approximately 10-fold above the basal fasting rate) [43]. Poor oral health, including tooth loss, commonly observed in older adults [44], can influence an individual’s dietary intake (avoiding harder foods such as fruits, raw vegetables, and meat, preferring cooked foods with softer textures), resulting in malnutrition [38]. Since different types of foods have distinct effects on gastric secretion, it is reasonable to assume that there are other factors that may impact gastric secretion in older individuals. Indeed, our Aging Lab group provided an in-depth analysis of the oral health care challenges and barriers for older adults, including time constraints, lack of training, inadequate resources, and poor collaboration among caregivers [45].

#### 4.2.2. Helicobacter Pylori

*H. pylori* infection is the most prevalent chronic bacterial infection in humans, with increased prevalence and severity associated with age [46]. Acute infection has been shown to result in hypochlorhydria through a number of mechanisms. These include direct inhibition of parietal cells and indirect inhibition of parietal cell function due to cytokine production, as well as hormonal, paracrine, and neural regulatory mechanisms [47,48,49]. Chronic infection may result in hypochlorhydria or hyperchlorhydria, depending on the severity and distribution of gastritis [50]. Most patients develop pangastritis and produce less than normal amounts of acid due to the functional inhibition of parietal cells, a process similar to that seen in acute infection [51]. This process is usually reversible and can be eradicated [52]. However, in patients with chronic persistent infection, atrophy of the oxyntic glands develops, resulting in the loss of parietal cells and consequent irreversible achlorhydria [53]. Studies in Japanese populations [21,22,24] have clearly demonstrated that acid secretion declines with aging in *H. pylori*-positive subjects. In 10–15% of infected patients, *H. pylori* infection may manifest as antral-predominant inflammation that reduces somatostatin production, which increases gastrin release and, subsequently, acid secretion [54]. These diverse responses to *H. pylori* infection may partially explain the different results of several studies with a small number of participants. Given the higher antibiotic resistance in the elderly, eradication failure may be more common in this population [55].

#### 4.2.3. Chronic Atrophic Gastritis

CAG is characterised by the loss of specialised cells within the gastric glands, such as parietal and chief cells, against a background of chronic inflammation [56]. This leads to a reduction or absence of gastric secretion products, such as IF, hydrochloric acid, and pepsinogen, and is significantly correlated with the severity of CAG [18]. It is generally associated with either *H. pylori* infection or autoimmunity. The prevalence of CAG ranges from 50% to 70% in people aged ≥ 60 years [57]. In an autopsy study, CAG was a common feature of the aging stomach in every second individual aged ≥ 70 years [58]. This phenomenon may explain some discrepancies in studies regarding age-related decline in gastric secretion, as most of them [8,9,10,15,16,19,20,23] did not consider the isolated effect of the histological condition of the gastric mucosa, which reduces gastric secretion, on gastric secretion. This may help clarify why, in countries like Japan, where the prevalence of CAG is higher, gastric secretion declines more sharply with age than in Western countries [24].

The postulated decrease in acid secretory capacity in older individuals may be secondary to the increased frequency and severity of atrophy associated with aging [17]. In studies where adjustment for histology was performed [17,18], age had no independent effect on gastric acid secretion. The rate of acid secretion may not change with aging unless there is a coexisting disease of the acid-secreting glandular mucosa, such as infection with *H. pylori* or CAG [59]. In this regard, stomach morphological status may be the most important determinant of gastric secretion at the population level. However, in one study [18], age was associated with reduced pepsin output.

#### 4.2.4. Antisecretory Therapies

PPIs are among the most prescribed drugs in developed countries, and elderly patients are particularly likely to be prescribed acid suppression [60]. They effectively block gastric acid secretion by irreversibly binding to and inhibiting the hydrogen-potassium ATPase residing on the luminal surface of the parietal cell membrane [61].

Histamine-2 receptor antagonists are an alternative therapy to PPIs, and they inhibit acid secretion by blocking the histamine receptor on parietal cells. Their use is now limited due to side effects and tachyphylaxis [62].

The widespread use of antisecretory drugs in the elderly population may account, at least in part, for the age-related decrease in gastric acid secretion [63,64].

### 4.3. Clinical Impact of Decline in Gastric Secretion

Gastric acid secretion serves several physiological functions beyond its protective role in ingested pathogens. It includes the digestion of proteins and absorption of micronutrients, such as iron, calcium, and vitamin B12, as well as medications like thyroid hormones. Additionally, it may play a role in preventing spontaneous bacterial peritonitis [59].

In humans, a decline in acid secretion may predispose patients to small intestinal bacterial overgrowth, which causes microscopic structural changes, particularly in older patients, including a decrease in mean villus height, mean crypt depth, and total mucosal thickness [65]. In older patients with chronic diarrhoea, small intestinal bacterial overgrowth and enteric infections should be considered in the differential diagnosis [66].

Gastric acid enhances iron solubility and intestinal iron absorption and facilitates the peptic digestion of dietary proteins bound to iron. Therefore, reduced acid secretion decreases bioavailable iron, leading to potential iron malabsorption and deficiency [67]. In addition, low gastric acid reduces the liberation of B12 bound to dietary proteins. Other vitamins (folate) or minerals (calcium, magnesium, and zinc) may be affected by diet and medicines, potentially leading to a broad spectrum of neurological, cognitive, psychiatric, and other clinical manifestations [68,69].

Hypochlorhydria or achlorhydria, regardless of the cause, leads to a compensatory increase in serum gastrin, a potent inducer of gastric epithelial cell proliferation and a risk factor for gastric cancer. Some patients, especially those with preneoplastic conditions such as atrophic gastritis, should be monitored using upper endoscopy [70,71]. A recent review demonstrated that the use of PPIs in older adults was associated with an elevated risk of osteoporotic-related fractures, Clostridioides difficile infection, community-acquired pneumonia, vitamin B12 deficiency, and kidney disease [72]. A systematic review revealed that older individuals who used PPIs exhibited a 15% increased risk of mortality from cancer and cardiovascular and renal diseases [73]. Due to their comorbidities, polypharmacy, and poor nutrition, this population is particularly vulnerable to the side effects of long-term treatment with PPIs [74]. However, prescribing antisecretory therapy in patients with possible hypochlorhydria or achlorhydria, such as older adults with atrophic gastritis, may not provide any clinical benefit. PPIs should be discontinued in patients without valid indications. When prescribed long-term, PPIs should be used at the lowest effective dose, and their need should be reassessed periodically [75].

IF is necessary for the optimal absorption of vitamin B12 [76]. Its physiological secretion far exceeds the amount required for cobalamin absorption. Thus, in patients with a generalised decline in gastric secretion, even low amounts of IF production is sufficient to prevent cobalamin deficiency, except in patients with pernicious anaemia [77]. Ardeman et al. [78] showed that a severe reduction of acid production is required before vitamin B12 absorption becomes impaired, and when acid production has fallen to zero, appreciable quantities of IF may still be produced.

Pepsin secretion has an important role in protein digestion. It works in concert with gastric acid in this process, although recent data suggest that pepsin may also play a role in killing ingested bacteria [79]. Pepsinogen is converted to the active protease pepsin at a low pH. Conversely, when pepsinogen is inactivated, pH rises above 4 [80]. Theoretically, hypochlorhydria/achlorhydria would impact protein digestion since pepsin would not be activated. In patients who have undergone partial or total gastrectomy, protein digestion may be impaired due to poor mixing with digestive secretions, and gastric pepsin deficiency may be contributory. However, in clinical practice, patients with hypochlorhydria/achlorhydria can still digest proteins, suggesting that proteolysis in the stomach is not essential for protein digestion [81]. Nevertheless, amino acids released during gastric digestion play a role in the release of cholecystokinin from duodenal and jejunal endocrine epithelial cells, which is critical for stimulating the release of pancreatic enzymes responsible for the digestion of all three macronutrients [82]. No studies have evaluated the role of impaired pepsin secretion in nutrient digestion and absorption in the elderly.

### 4.4. Strengths and Limitations

This scoping review was conducted according to the PRISMA, a rigorous and widely recommended guideline that increases robustness and reduces reporting errors. In addition, an extensive literature search was conducted using a meticulous, predefined protocol. To the best of our knowledge, this is the first review specifically designed to assess the influence of aging on gastric secretion. The inclusion of diverse geographic regions, encompassing studies from North America, Europe, and Asia, provides insights into the global patterns of the aging gastrointestinal tract. Moreover, this review provides a detailed exploration not only of the influence of aging *per se* but also regarding other aging-associated factors that may play a role in reducing gastric secretion.

However, this study has several limitations, primarily due to the inherent constraints of the included studies. The heterogeneity of the included works regarding the study population and methods of analysing gastric secretion makes it difficult to draw firm conclusions about the generalisability of the findings. Furthermore, the lack of standardised patient characteristics and potential factors not addressed in most studies that may influence gastric secretion (e.g., *H. pylori* status, CAG) makes it difficult to compare results, which reduces the power of the correlation analysis performed.

Future studies should focus on data representativeness and method standardisation to ensure more homogenous evidence-based results. Future research should prioritise longitudinal studies, larger cohorts, and histology-stratified analyses.

## 5. Conclusions

Early studies on gastric secretion suggest an age-related reduction. However, they preceded the recognition of *H. pylori* infection and disregarded the effect of CAG on secretory function and the impact of modern antisecretory drug therapy. Acid secretion may remain normal in elderly patients with normal gastric mucosa. However, several conditions associated with aging, including CAG, *H. pylori* infection, and the widespread use of PPIs, may contribute to the age-related decline in gastric secretion. Although the age-related decline in acid secretory function can be primarily attributed to age-associated disorders rather than direct aging, the impairment of secretory function appears to be very frequent in older individuals, and clinicians must consider this reduction, as it may impact their clinical practice. The same does not apply to IF, as even a significantly reduced IF secretion appears to be sufficient to preserve physiological function. Therefore, new studies are needed to clarify the impact of aging on gastric secretion.

## Figures and Tables

**Table 1:** Article identification and main results.

Authors (Year)	Study Characteristics	Patients	Aging Influence Outcomes			Comments
			Acid Secretion	Pepsin Secretion	Intrinsic Factor	
Khalil et al. (1988) [6]	Animal study	6 young rats (3 months old) 6 old rats (32 months old)	Decrease	Not evaluated	Not evaluated	▪ Small sample size
Maitra et al. (1988) [14]	Animal study	Young rats (4 months old) Old rats (21 months old)	Decrease	Decrease	Not evaluated	▪ No information on number of younger and older rats
Baron et al. (1963) [15]	Human study	40 patients (19–66 years old)	Decrease	Not evaluated	Not evaluated	▪ Small sample size ▪ No true older group
Andrews et al. (1967) [8]	Human study	24 patients (64–87 years old)	Decrease	Not evaluated	Decrease	▪ Small sample size ▪ No control/young group ▪ Assumed atrophic gastritis as a “normal” aging phenomenon
Borgström et al. (1973) [16]	Human study	221 patients < 60 years-old 31 patients > 60 years old	No change	Not evaluated	Not evaluated	▪ Only recruited patients with peptic ulcer disease
Kekki et al. (1982) [17]	Human study	437 patients > 14 years old	▪ Decrease (patients with atrophic gastritis) ▪ No change (males with normal gastric mucosa) ▪ Increase (females with normal gastric mucosa)	Not evaluated	Not evaluated	▪ Prospective study at the population level
Goldschmiedt et al. (1991) [9]	Human study	41 patients (23–71 years old)	▪ Increase in males ▪ Increase in females (no statistical difference)	Increase in males (*p* > 0.05) No difference in females	Not evaluated	▪ Small sample size ▪ No true older group
Katelaris et al. (1993) [10]	Human study	22 patients 18–30 years old 28 patients >65 years-old	No change	Not evaluated	Not evaluated	▪ Only recruited white males
Feldman et al. (1996) [18]	Human study	184 patients 18–64 years old 22 patients ≥ 65 years-old	▪ Decrease (patients with atrophic gastritis) ▪ No change (patients with normal gastric mucosa)	Decrease	Not evaluated	▪ Small elderly group
Hurwitz et al. (1997) [19]	Human study	248 patients ≥ 65 years old	No change	Not evaluated	Not evaluated	▪ No control/younger group ▪ Only recruited white patients
Trey et al. (1997) [20]	Human study	11 patients, 48–51 years old	Decrease	Not evaluated	Not evaluated	▪ Small sample size ▪ No true older group
Kinoshita et al. (1997) [21]	Human study	110 patients	▪ Decrease (*H. pylori*-negative patients); ▪ Decrease (*H. pylori*-positive patients)	Not evaluated	Not evaluated	▪ Prospective study in a Japanese population ▪ No information on the number of younger and older patients
Haruma et al. (2000) [22]	Human study	248 patients 16–64 years old 32 patients ≥ 65 years old	▪ No change (*H. pylori*-negative patients) ▪ Decrease (*H. pylori*-positive patients)	Not evaluated	Not evaluated	▪ Retrospective study in a Japanese population
Shih et al. (2003) [23]	Human study	645 patients < 65 years old 108 patients ≥ 65 years old	No change	Not evaluated	Not evaluated	▪ Retrospective study
Iijima et al. (2004) [24]	Human study	156 patients < 60 years old 57 patients > 60 years old	▪ No change/increase (*H. pylori*-negative patients) ▪ Decrease (*H. pylori*-positive patients)	Not evaluated	Not evaluated	▪ Prospective study in a Japanese population

## Data Availability

All data analysed in this review are included in this article. Further enquiries should be directed to the corresponding author.

## Supplementary Materials

The following supporting information can be downloaded at: https://www.mdpi.com/article/10.3390/biomedicines13071546/s1, Table S1: PRISMA-ScR checklist.

## Abbreviations

The following abbreviations are used in this manuscript:

CAG	Chronic atrophic gastritis
*H. pylori*	*Helicobacter pylori*
PPI	Proton pump inhibitor
